# Mapping the Landscape of Diaschisis in Traumatic Brain Injury: A Scoping Review

**DOI:** 10.1177/2689288X251379064

**Published:** 2025-10-06

**Authors:** Madison Evans, Cigdem Tosun, Ruchira M. Jha, Prajwal Ciryam, Volodymyr Gerzanich, J. Marc Simard

**Affiliations:** ^1^ Department of Neurosurgery, University of Maryland School of Medicine, Baltimore, Maryland, USA.; ^2^ Department of Neurology, Barrow Neurological Institute and St. Joseph’s Hospital and Medical Center, Phoenix, Arizona, USA.; ^3^ Department of Neurology, University of Maryland School of Medicine, Baltimore, Maryland, USA.; ^4^ Shock Trauma Neurocritical Care, Program in Trauma, R Adams Cowley Shock Trauma Center, University of Maryland Medical Center, Baltimore, Maryland, USA.; ^5^ Department of Pathology, University of Maryland School of Medicine, Baltimore, Maryland, USA.; ^6^ Department of Physiology, University of Maryland School of Medicine, Baltimore, Maryland, USA.

**Keywords:** brain mapping, diaschisis, functional recovery, neuroimaging, neural pathways, traumatic brain injury

## Abstract

Diaschisis is a phenomenon in which damage to one brain region leads to dysfunction in remote, yet functionally connected, areas. Although it has been well characterized in stroke, the complex, multifocal nature of traumatic brain injury (TBI) suggests that similar network-level disruptions could occur, yet the presence and impact of diaschisis in TBI remain underexplored. This gap may stem from a historical focus on cerebrovascular events, underrecognition of diaschisis in TBI, and methodological challenges related to TBI’s heterogeneous nature. This review maps diaschisis in TBI by examining models, mechanisms, neuroimaging, clinical features, and therapeutic interventions. A PRISMA-ScR guided search of PubMed, Embase, and Cochrane included studies explicitly addressing diaschisis in TBI from inception up to January 2025. Two independent reviewers screened titles, abstracts, and full texts, with discrepancies resolved by consensus. Twenty-three studies were included, encompassing 110 human participants, 497 animals, and one *in vitro* model. Among these, 57% used neuroimaging, 39% assessed functional outcomes, and 22% examined potential interventions. The predominant experimental model was rodent-controlled cortical impact, typically simulating moderate TBI. Contrarily, human studies were fewer and focused on severe TBI cases. Crossed cerebellar diaschisis was the most common neuroimaging finding (36%), with MRI used most frequently, followed by PET and SPECT. Across both clinical studies and preclinical models, key mechanisms of diaschisis included deafferentation, reduced metabolism, altered glutamate signaling, hypoperfusion, and distant apoptotic cell death. Motor deficits were more common with better recovery than cognitive impairments. Interventions such as MK-801 and Ifenprodil showed potential to reverse diaschisis, but others had limited effects. This review underscores the limited but growing understanding of diaschisis in TBI. Targeted research on mild-to-moderate TBI, interventions, and imaging-validation trials is needed to improve diagnosis and treatment.

## Introduction 

Diaschisis is a classical neurological phenomenon in which localized brain injury leads to functional deficits in regions distant from, but anatomically or functionally connected to, the primary lesion site. First conceptualized by Constantin von Monakow in the early 20th century, the term originally described the sudden loss of function in brain areas remote from a focal lesion, despite the absence of direct structural damage.^1^ Over time, the concept has evolved to encompass a variety of mechanisms, including disruption of functional connectivity, metabolic depression, and widespread network disintegration following focal or diffuse insults to the brain.^2^

Much of the foundational and contemporary research on diaschisis has emerged from studies of stroke. Within this well-characterized paradigm, several subtypes have been described, such as crossed cerebellar diaschisis (CCD), which refers to decreased metabolism and blood flow in the cerebellar hemisphere contralateral to a supratentorial lesion, and thalamocortical diaschisis, which involves disrupted functional connectivity and metabolic activity between the thalamus and cortical regions. These subtypes are supported by robust neuroimaging evidence from modalities such as positron emission tomography (PET), functional magnetic resonance imaging (fMRI), and diffusion tensor imaging (DTI).^2^ Stroke has thus served as a model for understanding how diaschisis contributes to remote dysfunction, informs recovery trajectories, and opens pathways for therapeutic intervention.

The clinical consequences of diaschisis in stroke illustrate why this phenomenon warrants closer attention. Diaschisis subtypes have been associated with motor incoordination, cognitive deficits, and impaired consciousness, symptoms that cannot be fully explained by the location of the primary lesion. For instance, patients with CCD exhibit significantly worse motor and cognitive outcomes during stroke recovery compared with matched controls (matched by age, stroke type, lesion laterality, and location), suggesting that remote cerebellar dysfunction contributes substantially to overall functional impairment.^3^ Similarly, thalamocortical diaschisis has been linked to widespread cortical hypometabolism following unilateral thalamic injury, correlating to global neuropsychological deficits.^4^

These manifestations reflect disruptions in neural connectivity and metabolic activity in regions spatially remote but functionally integrated with the lesion site, complicating both diagnosis and prognosis. If unrecognized, the effects of diaschisis may lead to underestimation of injury severity or missed opportunities for targeted rehabilitation. As neuroimaging advances continue to illuminate the brain’s network-level responses to focal insults, diaschisis provides a critical framework for understanding and intervening in these widespread functional impairments.

The established role of diaschisis in stroke underscores the need for further research across injury types, particularly in traumatic brain injury (TBI), where similar mechanisms of remote dysfunction may exist but remain poorly characterized and notably underdeveloped. This underrepresentation persists despite TBI’s potential for widespread neural disruption, including both focal lesions and diffuse pathologies such as axonal shearing and multifocal contusions.^5^ Contributing factors may include a historical research bias toward stroke, the clinical and pathological heterogeneity of TBI, and technical limitations in capturing dynamic, network-level dysfunction across time and injury severity in TBI populations.^6^ Understanding diaschisis is essential not only for optimizing clinical care in diverse brain injuries, but also for advancing a systems-based view of brain dysfunction and recovery that transcends traditional lesion-focused models.

This knowledge gap underscores the need for a comprehensive synthesis of how diaschisis is defined, investigated, and interpreted within TBI research. A systematic mapping of this emerging field is critical not only for clarifying terminology and methodologies but also for informing translational efforts to optimize diagnosis, prognosis, and rehabilitation. This scoping review aims to map the existing literature on diaschisis in TBI, identify its neurobiological and clinical characterizations, and highlight opportunities for advancing research and care.

## Methods

### Design

This study was designed as a scoping review of the literature, focusing on diaschisis in the context of TBI by examining models, mechanisms, neuroimaging, functional outcomes, and/or interventions. It was conducted following the guidelines of PRISMA-ScR (Preferred Reporting Items for Systematic Reviews and Meta-Analyses Extension for Scoping Reviews).^7^ No formal review protocol was developed or registered for this scoping review.

### Search strategy

A comprehensive literature search was conducted using PubMed, Embase, and Cochrane databases to identify studies that explicitly addressed diaschisis models, mechanisms, neuroimaging, functional outcomes, and/or interventions. The search strategy used only the term “diaschisis” without specifying TBI; studies were subsequently screened and selected based on inclusion and exclusion criteria that focused specifically on TBI-related contexts. These databases were searched from inception to January 2025, with the most recent search executed in January 2025. Search terms for each database were as follows:

#### PubMed

(“diaschis*”[Title/Abstract] OR “Diaschisis”[MeSH Terms])

#### Embase

(‘diaschisis’/exp OR ‘diaschises’:ti,ab OR ‘diaschisis’:ti,ab)

#### Cochrane

(Diaschisis)

### Eligibility criteria

**FIG. 1.**
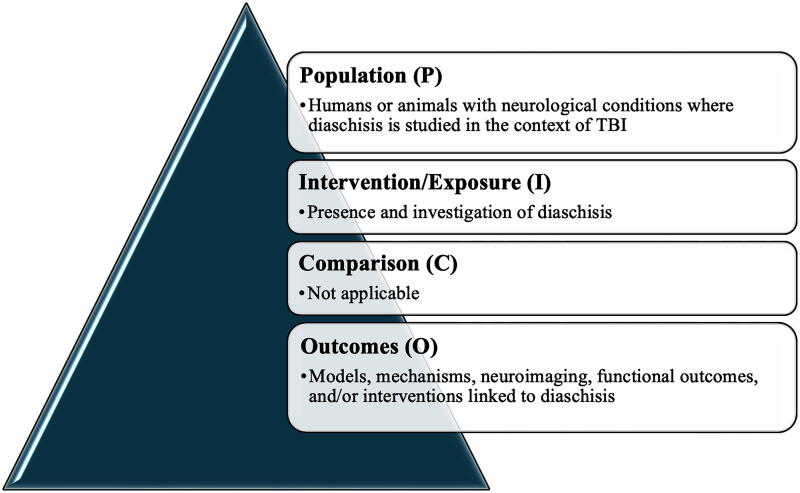
PICO framework. This scoping review was guided by the PICO framework to define the population, intervention/exposure, comparison, and outcomes of interest. The population included humans or animals with neurological conditions where diaschisis was studied in the context of traumatic brain injury (TBI). The intervention/exposure focused on the presence and investigation of diaschisis. Comparison was not applicable in this review. Outcomes of interest included models, mechanisms, neuroimaging findings, functional outcomes, and interventions linked to diaschisis.

This scoping review was guided by the PICO framework to define the population, interventions, and outcomes of interest (Fig. 1). Eligible studies had to explicitly address diaschisis models, mechanisms, neuroimaging, functional outcomes, and/or interventions in the context of TBI.

Both human and animal studies were eligible for inclusion. In human studies, there were no restrictions based on age, cause, severity, or chronicity of TBI. Studies were included if TBI was self-reported; medical documentation or formal clinical confirmation of TBI was not required. Studies that included mixed populations of acquired brain injury (ABI), such as stroke and TBI, were eligible only if results pertaining to TBI were presented separately.

For animal studies, all TBI models were considered acceptable. To be included, animal studies had to contribute translational or clinically relevant insights, defined as findings that could be mapped to one or more of the diaschisis domains of interest: models, mechanisms, neuroimaging, functional outcomes, or therapeutic interventions.

Eligible study designs included randomized controlled trials, prospective and retrospective cohort studies, chart reviews, case studies or series, cadaveric or anatomical studies, and both narrative and systematic literature reviews. Only peer-reviewed articles published in English or in other languages with accessible English translations were considered.

Studies were excluded if they did not explicitly mention diaschisis, focused solely on unrelated neurological phenomena, were limited to conference abstracts lacking sufficient methodological or outcome detail, or were animal studies that did not demonstrate relevance to diaschisis within the defined domains.

### Study selection and screening process

Two reviewers independently screened all titles, keywords, and abstracts for relevance based on predefined inclusion and exclusion criteria. Full texts of potentially eligible studies were then reviewed to determine final eligibility. All discrepancies were resolved through discussion and consensus.

### Data extraction

A standardized data extraction template was used to collect the following information from each included study: first author, year, country, study design, study type, sample size, population, TBI severity, and injury location. Further data were collected regarding diaschisis model type, neuroimaging findings, mechanisms of diaschisis, functional outcomes, and interventions used. Data extraction was performed independently by two reviewers to ensure accuracy and consistency. Discrepancies were resolved through discussion and consensus.

### Data synthesis

All extracted data were organized into standardized charting tables to facilitate comparison across studies. Descriptive synthesis was conducted by the review team through group discussions to identify common patterns, trends, and gaps in the literature. Where appropriate, visual representations such as bar charts and pie charts were created to summarize key study characteristics. However, the primary method of data presentation was through comprehensive tables reflecting the extracted information on diaschisis models, mechanisms, neuroimaging findings, functional outcomes, and interventions.

## Results

### Study selection

**FIG. 2.**
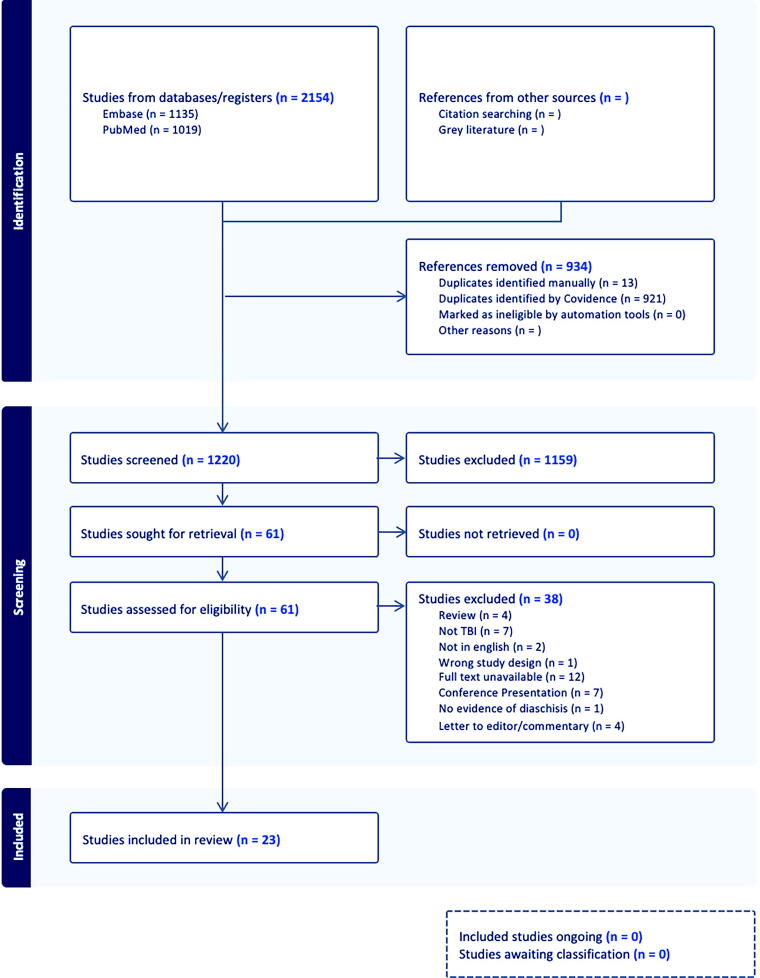
PRISMA flow diagram. Two independent reviewers screened all titles and abstracts for relevance. Full texts of potentially eligible articles were obtained and assessed against inclusion and exclusion criteria, with discrepancies resolved by consensus. The initial pool of 2,154 studies was reduced by removing 934 duplicates, leaving 1,220 studies. After abstract screening, 1,159 studies were excluded. In the second phase, 61 full-text articles were reviewed, and 23 studies were deemed appropriate for inclusion in the final review.

A total of 2,154 records were identified through database searches. After removal of duplicates (*n* = 934), 1,220 unique studies remained for abstract screening. Of these, 1,159 were excluded. The full texts of the remaining 61 studies were assessed in detail, and 23 articles met all eligibility criteria and were included in the final review (Fig. 2).

### Study characteristics

**FIG. 3.**
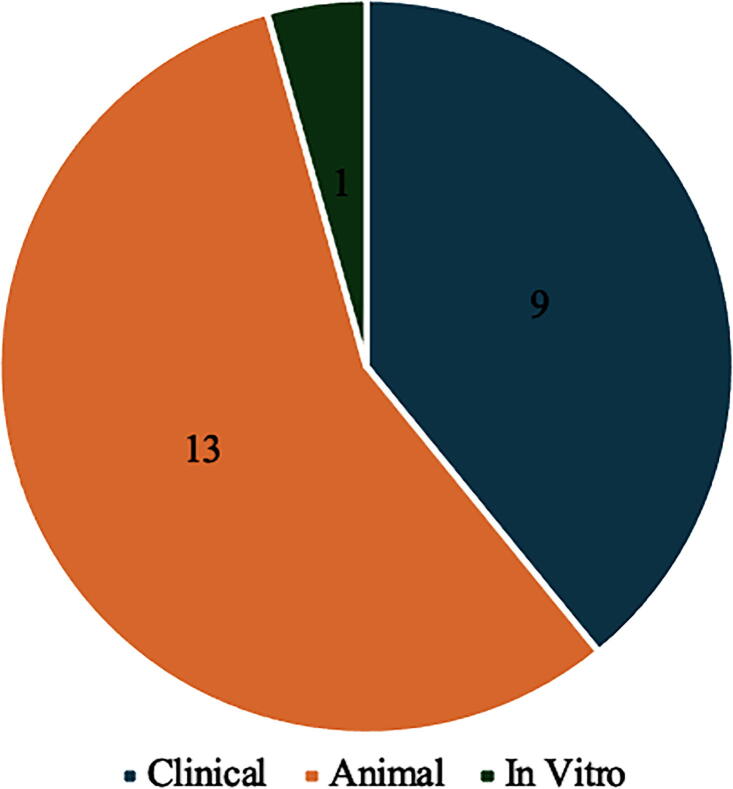
Distribution of study type. The figure highlights a predominance of preclinical studies, including 13 animal studies and one *in vitro* neuronal model. The remaining nine studies were clinical, mostly involving patients with moderate-to-severe traumatic brain injury.

**FIG. 4.**
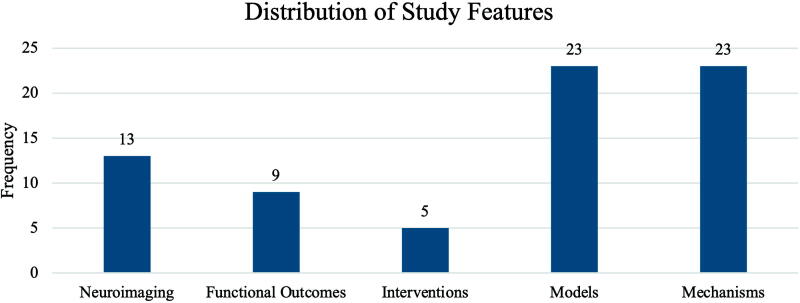
Distribution of study features. This figure illustrates the distribution of study features among the included articles. All studies investigated mechanisms and models of diaschisis (*n* = 23), while several explored neuroimaging correlates (*n* = 13), functional outcomes (*n* = 9), and interventions (*n* = 5).

The 23 included studies comprised a diverse range of methodologies and populations, with sample sizes ranging from single-subject case reports^8–11^ to large experimental groups of up to 81 subjects^12^ (Table 1). Figure 3 highlights a predominance of preclinical studies, including animal studies (*n* = 13)^12,14,16–18,20,21,23,24,26,28–30^ and one *in vitro* neuronal model.^15^ The remainder were clinical studies (*n* = 9),^8–11,13,19,22,25,27^ mostly involving patients with moderate-to-severe TBI. Across the 23 studies, research was conducted in eight countries, including the United States, Germany, the United Kingdom, France, the Czech Republic, Taiwan, Australia, Korea, and the United Arab Emirates. Figure 4 illustrates the distribution of study features, revealing that all studies investigated mechanisms and models of diaschisis, with several exploring neuroimaging correlates (*n* = 13),^8–10,12–14,19,22,25–27^ functional outcomes (*n* = 9),^8–10,12,14,19,22,24,29^ and interventions (*n* = 5).^15,16,22,26,29^

**Table 1.** Summary of Study Characteristics

Author (year)	Study type	Design	Sample size	Population	TBI severity	Injury location	Country
Alavi (1997)^13^	Clinical	Longitudinal	29	Human	Severe	Multiple (Case Series)	USA
Ali (2015)^8^	Clinical	Longitudinal	1	Human	Severe	Right hemisphere	UAE
Boggs (2024)^14^	Animal	Experimental	23	Rats	Mild	Left primary somatosensory cortex	USA
Deleglise (2018)^15^	In Vitro	Experimental	N/A	Neuronal culture	N/A	Cortico-striatal network	France
Derakhshan (2009)^9^	Clinical	Longitudinal	1	Human	Severe	Right hemisphere	USA
Drubach (1994)^10^	Clinical	Longitudinal	1	Human	Severe	Right parieto-occipital region	USA
Ihbe (2022)^16^	Animal	Experimental	54	Mice	Moderate	Sensorimotor cortex	Germany
Imbrosci (2015)^17^	Animal	Experimental	67	Rats	Mild–Moderate	Visual cortex	Germany
Joashi (1999)^18^	Animal	Experimental	9	Rats	Moderate	Frontoparietal cortex	UK
Kaech (1996)^19^	Clinical	Longitudinal	10	Human	Severe	Multiple (Case Series)	Czech Republic
Kempinsky (1958)^20^	Animal	Experimental	Not specified	Cats	Moderate–Severe	Optic cortex	USA
Le Prieult (2017)^21^	Animal	Experimental	80	Mice	Moderate	Parietal cortex	Germany
Lin et al. (2023)^22^	Clinical	Longitudinal	30	Human	Mild–Moderate	Not available	Taiwan
Martins et al. (1977)^23^	Animal	Experimental	20	Macaques	Focal lacerating injury	Superior frontal gyrus	USA
Nishibe et al. (2010)^24^	Animal	Experimental	24	Rats	Moderate	Motor cortex (caudal forelimb area)	USA
Poudel et al. (2020)^25^	Clinical	Longitudinal	36	Human (Adolescents)	Moderate–Severe	Varies between individuals	Australia
Schmitt et al. (1998)^26^	Animal	Experimental	30	Rats	Mild	Optic nerve	USA
Simard et al. (2025)^12^	Animal	Experimental	81	Mice	Moderate	Temporal lobe	USA
Sztriha et al. (1996)^27^	Clinical	Longitudinal	2	Human (Children)	Not specified	Not specified	UAE
Taylor et al. (2006)^28^	Animal	Experimental	24	Rats	Needle injury	Forebrain or cerebellum	UK
Verley et al. (2019)^29^	Animal	Experimental	21	Rats	Moderate	Parietal-temporal cortex	USA
Wiley et al. (2016)^30^	Animal	Experimental	64	Mice	Mild–Severe	Parietal-temporal cortex	USA
Yang et al. (2014)^11^	Clinical	Longitudinal	1	Human	Moderate–Severe	Occipital lobe	Korea

N/A, not applicable.

### Models of diaschisis

The included studies used a range of experimental and clinical models to investigate diaschisis following TBI (Table 2). Nine studies used human clinical frameworks or case reports. These included observational imaging studies in patients with moderate-to-severe TBI,^10,13,19,22,25^ single-case clinical descriptions involving subdural hematoma or long-term seizure sequelae,^8,9,11^ and a pediatric imaging study assessing diaschisis in children with hemiplegia of various traumatic etiologies.^27^ One study used a computational imaging model to simulate structural degeneration patterns using diffusion-weighted and anatomical MRI.^25^

**Table 2.** Models of Diaschisis

Author (year)	Model type	Description	Key features	Strengths	Limitations
Alavi (1997)^13^	Human Clinical Study	CCD in TBI patients	FDG-PET vs CT/MRI in 19 patients	- Human relevance - Identifies cerebellar hypometabolism	- Small sample - Observational - No experimental control
Ali (2015)^8^	Clinical Case Study	Severe TBI with seizures	5-year post-TBI, left-sided seizures	- Long-term outcome insights - Links epilepsy and cortical injury	- Single case - No control group - Low generalizability
Boggs (2024)^14^	Rat TBI Model	CCI	Left S1 cortex, pneumatic impactor	- High control over injury parameters - Relevant for diaschisis research	- Limited to cortical injury - Rodent model limits human applicability
Deleglise (2018)^15^	In Vitro Model	Cortico-striatal co-culture	Microfluidic glutamate injury, NMDAR activation	- High experimental precision -Cellular mechanisms of diaschisis	- *In vitro* only - Lacks systemic interactions
Derakhshan (2009)^9^	Clinical Case Study	Subdural hematoma with disconnection syndrome	Right-sided hematoma, transcallosal effects	- Highlights hemispheric disconnection - Clinical relevance	- Single case - No control
Drubach (1994)^10^	Clinical Framework	Severe TBI with visual symptoms	SPECT imaging, parieto-occipital hypoperfusion	- Real-world clinical imaging - Direct human application	- Poor variable control - Complex to isolate diaschisis effects
Ihbe (2022)^16^	Mouse TBI Model	CCI	Sensorimotor cortex injury	- Reproducible - Controlled injury size	- Rodent-specific - Limited injury area
Imbrosci (2015)^17^	Rat TBI Model	Focal cortical laser lesions	Induces precise lesions	- High reproducibility	- Not directly generalizable to humans
Joashi (1999)^18^	Needle Injury Model	Traumatic forebrain needle injury	P14 rats, mimics infarct	- Controlled depth/site - Pediatric analog	- Focal-only - Not representative of diffuse injury
Kaech (1996)^19^	Clinical Neuroimaging	PET metabolic study in TBI	Identifies diaschisis, CCD, cortical hypometabolism	- *In vivo* metabolic data - Demonstrates CCD in TBI	- May not reflect cognitive/behavioral outcomes
Kempinsky (1958)^20^	Feline Model	Focal cortical damage	Various methods (MCA occlusion, CO_2_, cautery, suction)	- Gyrencephalic brain - Interhemispheric analysis	- Non-human - Small sample - Historical methods
Le Prieult (2017)^21^	Mouse TBI Model	Focal CCI in mice	Mimics cortical diaschisis	- High reproducibility	- Rodent-only - Generalizability to human injury limited
Lin et al. (2023)^22^	Clinical Study	iPBM in mild — moderate TBI	SPECT pre/post-treatment	- Human relevance - Clinical intervention model	- No long-term data - Small, male-dominant sample - Optimization needed
Martins et al. (1977)^23^	Monkey Model	Focal air blast injury	Measures CBF, O_2_, consumption	- Primate relevance - Real-time physiology monitoring	- Anesthesia effects - Short (4 h) study window - Small sample
Nishibe et al. (2010)^24^	Rat CCI Model	Focal cortical impact	Persistent motor deficit	- Reproducible injury model	- Diaschisis not clearly observed
Poudel et al. (2020)^25^	Clinical Imaging Model	Gray matter thinning simulations post-TBI	PCA + MRI/DWI in adolescents	- Individualized network analysis - Detects progressive damage	- Small n (17)-High imaging needs - Limited acute phase use
Schmitt et al. (1998)^26^	Optic Nerve Model	Nerve crush/cut recovery model	Visual cortex metabolism post-injury	- Tracks intrinsic vs extrinsic recovery - Metabolic mapping	- Vision-specific - Indirect measure (LCGU) - Short follow-up
Simard et al. (2025)^12^	Mouse tlCont Model	Temporal lobe hemorrhagic injury	EEG, memory, anxiety, IHC over 7–35 days	- Mimics human tlCont - Captures chronic & emotional outcomes	- Limited spatial access - No longitudinal imaging
Sztriha et al. (1996)^27^	Pediatric SPECT Model	Hemiplegia with diaschisis	99mTc-HMPAO SPECT in 14 children	- Multi-etiology insights - Pediatric-focused diaschisis patterns	- Cross-sectional -Small sample - Limited perfusion-symptom correlation
Taylor et al. (2006)^28^	Neonatal Diaschisis Model	Apoptosis after forebrain injury	P14 rats, HI or needle injury, EM, PARP	- Cell-level insight - Multi-method validation	- Neonatal brain only - No functional testing
Verley et al. (2019)^29^	Diaschisis Recovery Model	fMRI + silencing post-CCI	Muscimol at 1 vs 4 weeks, behavioral testing	- Rehab window identified - Links circuitry to behavior	- Not clinically replicable - Effects vary by timing
Wiley et al. (2016)^30^	Mouse Diaschisis Model	Anatomical spread post-TBI	EM, MAP-2, silver stain in contralateral cortex/thalamus	- High-resolution (EM + histo) - Shows reversible damage	- Silver stain clinical relevance unclear - Lacks behavioral analysis
Yang et al. (2014)^11^	Clinical Case	Chronic TBI with CCD and hemianopia	PET + DTI, occipital hypometabolism	- Multimodal chronic-stage imaging - Demonstrates CCD functionally	- Single case - No comparative modeling

CCD, crossed cerebellar diaschisis; FDG-PET, fluorodeoxyglucose positron emission tomography; CT, computed tomography; MRI, magnetic resonance imaging; CCI, controlled cortical impact; NMDAR, *N*-methyl-d-aspartate receptor; SPECT, single photon emission computed tomography; P14, postnatal day 14; MCA, middle cerebral artery; CO_2_, carbon dioxide; iPBM, intracranial photobiomodulation; CBF, cerebral blood flow; O_2_, oxygen; PCA, principal component analysis; DWI, diffusion-weighted imaging; LCGU, local cerebral glucose utilization; tlCont, temporal lobe contusion; EEG, electroencephalography; IHC, immunohistochemistry; 99mTc-HMPAO, technetium-99m hexamethylpropyleneamine oxime; HI, hypoxia-ischemia; EM, electron microscopy; PARP, poly (ADP-ribose) polymerase; fMRI, functional magnetic resonance imaging; MAP-2, microtubule-associated protein 2; DTI, diffusion tensor imaging.

Eleven studies used rodent models to induce focal brain injuries and assess remote effects indicative of diaschisis. These included controlled cortical impact (CCI) paradigms in rats or mice,^12,14,16,21,24,29,30^ laser-induced cortical lesions,^17^ traumatic needle injury in developing rats,^18,28^ and optic nerve crush or cut to examine metabolic recovery and extrinsic modulation.^26^ Within this group, a neonatal rat model of forebrain injury was used to examine remote cerebellar apoptosis, representing diaschisis in the developing brain,^28^ while a mouse model of hemorrhagic temporal lobe contusion investigated both behavioral outcomes and interhemispheric diaschisis using a combination of sensorimotor testing, electroencephalogram (EEG), and immunohistochemistry.^12^

Two studies used nonrodent animal models. Martins and Doyle^23^ applied a blast-induced focal laceration model in monkeys to measure cerebral blood flow and oxygen consumption, while Kempinsky^20^ used unilateral injury in cats induced via multiple methods, including cautery and suction, to induce focal injury and evaluate interhemispheric connectivity. In addition, one study used an *in vitro* model consisting of a reconstructed cortico-striatal network in microfluidic chambers to explore excitotoxic mechanisms related to diaschisis.^15^

### Mechanisms

The included studies described various mechanisms of diaschisis following TBI, often involving disrupted metabolic, structural, and neurochemical signaling in areas distant from the primary lesion (Table 3). Some mechanisms reflect upstream drivers of diaschisis, such as disrupted structural connectivity and neurotransmitter imbalance, while others represent downstream consequences of cellular dysfunction in remote areas. In addition, evidence synthesized in this review highlights the role of non-neuronal cells and neurovascular dysfunction in mediating or sustaining diaschitic effects.

**Table 3.** Mechanisms of Diaschisis

First author (year)	Mechanism	Description	Affected regions	Neurochemical/ molecular findings	Key evidence
Alavi (1997)^13^	Crossed cerebellar diaschisis	Cerebellar impairment due to disrupted excitatory cortical/extraparenchymal input	Cerebral cortex, contralateral cerebellum	Disrupted corticopontine fibers; possible glutamate and GABA involvement	40% of focal unilateral lesions; more frequent in cortical/extraparenchymal injuries
Ali (2015)^8^	Crossed diaschisis (seizure-induced)	Metabolic/structural changes with hemispheric edema	Right cortex, contralateral cerebellum	↑ Glutamate/Aspartate/Lactate/CBF; ↓ NAA	EEG: right hemisphere seizures; imaging: panhemispheric edema; improved with AEDs
Boggs (2024)^14^	Crossed diaschisis (TBI)	Remote impairment due to TBI-induced deafferentation	Cortex, striatum, thalamus	↓ Glutamate/GSH/NAA; ↑ Aspartate/Lactate	CBF/metabolic changes ipsilateral and contralateral to lesion
Deleglise (2018)^15^	Trans-synaptic degeneration	Glutamate-mediated spread of dysfunction	Cortex, striatum	↑ GluN2B-NMDAR activation	In vitro: axotomy/ischemia models
Derakhshan (2009)^9^	Transcallosal diaschisis	Temporary left-hemisphere deafferentation	Right hemisphere, corpus callosum	Impaired interhemispheric signaling	Neuropsych tests, DWI, bimanual tasks
Drubach (1994)^10^	Transhemispheric diaschisis	Focal injury with remote suppression	Bilateral occipital cortex	↓ CBF and metabolism	SPECT: decreased occipital activity post-TBI
Ihbe (2022)^16^	GABAergic dysregulation	VGCC subunit switch in SST interneurons	Contralateral sensorimotor cortex	↑ CaV1.3, ↓ CaV1.2; altered GABA signaling	Rodent TBI model, electrophysiology, isradipine reversal
Imbrosci (2015)^17^	Transhemispheric diaschisis	Neuronal hyperexcitability in undamaged cortex	Contralateral cortex	↑ Excitability, E/I imbalance	In vivo/ex vivo rat models, patch-clamp
Joashi (1999)^18^	Apoptosis	Remote cell death post-HI/TBI	Hippocampus, cerebellum	↑ PARP cleavage, apoptosis markers	Neonatal rat models, HI and trauma
Kaech (1996)^19^	Hypometabolism	Decreased metabolism in functionally connected sites	Frontal/parieto-occipital cortex, cerebellum	↓ FDG uptake	PET: chronic TBI patients
Kempinsky (1958)^20^	Cerebral shock (diaschisis)	Transient remote suppression from focal lesions	Cerebral cortex, connected regions	Suppressed neural activity; non-vascular	Animal models: electrical depression based on lesion type
Le Prieult (2017)^21^	Transhemispheric diaschisis	Contralateral hyperactivity from unilateral injury	Motor/somatosensory cortex	Impaired phasic GABA; ↑ tonic GABA-A subunits	Electrophysiology 24–48 h post-TBI
Lin et al. (2023)^22^	Mitochondrial proliferation	Functional regeneration post-TBI	Cortex, cerebellum	↓ Oxidative stress	SPECT in TBI patients
Martins (1977)^23^	Posttraumatic diaschisis	Focal trauma alters contralateral regions	Bilateral superior frontal gyri	Not applicable	Macaque air-jet injury: slight CBF reduction
Nishibe (2010)^24^	Diaschisis-like effect	RFA dysfunction despite structural preservation	Primary motor cortex (CFA), premotor cortex (RFA)	Implicated altered corticocortical signaling	Rats: CCI impaired RFA motor maps
Poudel (2020)^25^	Network degeneration	Gray matter loss via network deafferentation	Hippocampus, temporal cortex, striatum	Wallerian degeneration; protein aggregation	Adolescents with TBI: NDM predicted chronic atrophy
Schmitt (1998)^26^	Visual diaschisis	Reversible suppression after optic injury	Contralateral visual cortex	ACh role (physostigmine)	LCGU unchanged post-optic nerve cut/crush
Simard (2025)^12^	Temporal lobe contusion	Apoptosis, gliosis, axonal degeneration in transcallosal fibers	Temporal lobe, corpus callosum	↓ PV+ GABAergic neurons; gliosis	Mice: persistent memory deficits, bilateral pathology
Sztriha (1996)^27^	Cerebral diaschisis	Perfusion deficits remote from lesion	Cortex, cerebellum, thalamus, BG	Disrupted corticocerebellar/thalamocortical pathways	SPECT: 14 pediatric cases with perfusion deficits
Taylor (2006)^28^	Cellular diaschisis	Remote apoptosis from forebrain injury	Hippocampus, cerebellum	↑ PARP cleavage, apoptotic markers	Neonatal rats: cerebellar apoptosis post-forebrain HI
Verley (2019)^29^	Diaschisis from CCI	Remote motor cortex dysfunction via CCI	Ipsilesional/contralesional motor cortex	GABA system (muscimol-sensitive)	TBI rats: dysfunction reversed by neuromodulation
Wiley (2016)^30^	Diaschisis	Reactive gliosis and degeneration in remote regions	Contralateral cortex, thalamus, cerebellum	↓ Neurofilaments; gliosis; WFA loss; intact MAP-2	Histology: widespread degeneration, EM: membrane intact
Yang (2014)^11^	Diaschisis	Occipital injury → chronic hypometabolism/CCD	Occipital lobe, cerebellum, optic tracts	CBF suppression via corticopontocerebellar tract	PET/DTI: CCD and optic radiation injury

GABA, gamma-aminobutyric acid; NAA, *N*-acetylaspartate; AED, antiepileptic drug; GSH, glutathione; GluN2B-NMDAR, glutamate [*N*-methyl-d-aspartate] receptor subtype gluN2B; VGCC, voltage-gated calcium channel; SST, somatostatin; CaV1.3, calcium voltage-gated channel subunit alpha1 D; CaV1.2, calcium voltage-gated channel subunit alpha1 C; E/I, excitatory/inhibitory; GABA-A, gamma-aminobutyric acid type A receptor; RFA, rostral forelimb area; CFA, caudal forelimb area; NDM, network diffusion model; ACh, acetylcholine; PV+, parvalbumin-positive; BG, basal ganglia; WFA, Wisteria floribunda agglutinin.

Upstream drivers of diaschisis often involve structural disconnection and disruptions in excitatory–inhibitory neurotransmission. For instance, CCD was observed in association with cortical or extraparenchymal injury, disrupted corticopontine tracts, and altered glutamate/gamma-aminobutyric acid (GABA) signaling.^13^ Diaschisis-like suppression of premotor areas following primary motor cortex injury suggested impaired corticocortical signaling.^24^ Additional studies described trans-synaptic degeneration involving glutamatergic hyperactivity in cortico-striatal pathways,^15^ as well as impaired interhemispheric coordination resulting from transcallosal disconnection.^9^ Persistent hypometabolism and CCD were identified after occipital injury, with involvement of cortico-ponto-cerebellar tracts.^11^

Downstream patterns of diaschisis were frequently reported as metabolic disturbances, altered cerebral blood flow, and impaired coordination in areas distant from the lesion. Seizure-induced diaschisis, for example, was linked to elevated glutamate and aspartate, reduced *N*-acetylaspartate (NAA), and increased cerebral blood flow.^8^ Traumatic diaschisis showed metabolic changes in glutamate, aspartate, glutathione, lactate, and NAA across multiple regions.^14^ Reduced occipital metabolism from focal injury was consistent with transhemispheric diaschisis.^10^ Several studies also highlighted altered inhibitory control. GABAergic dysregulation in the contralateral cortex resulted from post-TBI shifts in expression of the calcium channels, CaV1.2 and CaV1.3, in somatostatin-expressing (SST) interneurons,^16^ and heightened excitability was reported in the intact hemisphere after injury.^17^ Transient cortical suppression, or “cerebral shock,” was seen in animal models of focal lesions.^20^ Impaired phasic GABA transmission and hyperexcitability were found in the contralateral cortex post-injury.^21^ Apoptotic cell death occurred in remote hippocampal and cerebellar regions after early injury,^18^ and chronic hypometabolism was observed in structurally intact cortex and cerebellum.^19^ Functional diaschisis in bilateral motor cortices was reversed with GABAergic neuromodulation.^29^

Gliotic changes further support the involvement of nonneuronal cells, including astrocytes and microglia, in sustaining or amplifying diaschitic dysfunction over time. Temporal lobe contusion caused memory deficits, interneuron loss, and bilateral glial activation with notable microglia, astrocyte, and oligodendrocyte involvement.^12^ Diffuse gliosis and degeneration in remote cortical and subcortical regions were observed in chronic TBI.^30^ In addition, apoptotic cerebellar damage, mediated in part by microglia-driven inflammation and astrocytic dysfunction, followed forebrain injury in neonatal rats.^28^ Pediatric diaschisis involved neurovascular unit dysfunction, evidenced by perfusion deficits across multiple deep brain structures.^27^

Other mechanisms included mitochondrial proliferation and reduced oxidative stress in remote brain regions,^22^ and network degeneration across cortico-striatal and temporal circuits driven by deafferentation.^25^ Visual system diaschisis showed contralateral suppression not reversed by cholinergic stimulation.^26^

### Neuroimaging

Neuroimaging studies revealed distinct patterns of diaschisis in TBI, marked by structural and metabolic alterations in regions remote from the primary lesion (Table 4). CCD was commonly reported and characterized by contralateral cerebellar hypometabolism or hypoperfusion following cortical injury. Alavi et al.^13^ reported CCD via fluorodeoxyglucose PET (FDG-PET) with both contralateral and ipsilateral cerebellar hypometabolism in the chronic stages of TBI. Similarly, Ali et al.^8^ reported right pancortical and contralateral cerebellar diffusion restriction on diffusion weighted imaging (DWI) with corresponding fluid-attenuated inversion recovery (FLAIR) hyperintensities and edema, along with vascular dilation on MRA five years after TBI. CCD was further supported by findings from Sztriha et al.^27^ using single photon emission computed tomography (SPECT), showing contralateral cerebellar perfusion deficits in children, and from Yang et al.^11^ who also identified contralateral cerebellar hypometabolism and disrupted corticopontocerebellar tracts and ipsilateral optic radiations with PET and DTI. Lin et al.^22^ reported increased cerebellar perfusion between baseline (within 3 weeks prior to intravascular photobiomodulation [iPBM] initiation) and post-treatment follow-up after three courses over 2–3 months, suggesting a potential reduction in diaschisis with chronicity.

**Table 4.** Neuroimaging Findings of Diaschisis

First author (year)	Type of diaschisis	Neuroimaging modality	Key imaging findings	Timing Post-TBI
Alavi (1997)^13^	Crossed Cerebellar Diaschisis	FDG-PET, CT, MRI	Contralateral and ipsilateral cerebellar hypometabolism in focal unilateral lesions	Chronic stages (variable)
Ali (2015)^8^	Crossed Cerebellar Diaschisis	MRI (DWI, FLAIR), MRA	Right pancortical DWI restriction, FLAIR hyperintensities, left cerebellar involvement, arterial dilation	5 years
Boggs (2024)^14^	Cortical-cortical	MRI (T2-RARE)	Peak lesion volume on Day 2, followed by progressive decrease	1–14 days
Derakhshan (2009)^9^	Cortical-cortical	Diffusion-Weighted Imaging (DWI)	Thin isodense subdural hematoma over right hemisphere, accentuated occipitally; no contralateral extension	2 weeks
Drubach (1994)^10^	Transhemispheric	SPECT	Decreased radiotracer uptake in occipital lobes, right > left	3 weeks
Kaech (1996)^19^	Subcortical-cortical	PET	Mildly reduced metabolism in frontal and parieto-occipital cortex	18 days–62 months
Lin et al. (2023)^22^	Crossed Cerebellar Diaschisis	SPECT	Increased cerebellar perfusion, reduced CCD	Up to 3 years
Poudel et al. (2020)^25^	Wallerian Degeneration	MRI (Connectome Reconstruction)	Predicts individual atrophy patterns and timing	Up to 5 years
Schmitt et al. (1998)^26^	Visual Diaschisis	Local Cerebral Glucose Utilization (LCGU)	No changes in contralateral visual projection areas; physostigmine ineffective	2 and 9 days post-injury
Simard et al. (2025)^12^	Interhemispheric, Transcallosal	EEG	Seizure activity	7–21 days
Sztriha et al. (1996)^27^	Crossed Cerebellar Diaschisis	99mTc-HMPAO SPECT	Hypoperfusion in contralateral cerebellum	Later, unspecified
Verley et al. (2019)^29^	Functional Diaschisis	fMRI	Decreased activation in contralesional cortex post-muscimol; baseline activation before	5–6 weeks
Yang et al. (2014)^11^	Crossed Cerebellar Diaschisis (CCD)	18FDG-PET, DTI	Hypometabolism in unilateral occipital lobe and contralateral cerebellum; disruption of ipsilateral optic radiations	4 years

FLAIR, fluid-attenuated inversion recovery; MRA, magnetic resonance angiography; T2-RARE, T2-weighted rapid acquisition with relaxation enhancement; 18FDG-PET, fluorine-18 fluorodeoxyglucose positron emission tomography.

Cortical-cortical and transcallosal diaschisis were also identified using a variety of imaging modalities. Boggs et al.^14^ observed evolving cortical lesions on MRI over a 14-day period, with remote cortical involvement consistent with cortical-cortical diaschisis, noting peak lesion volume early after injury. Drubach et al.^10^ detected decreased radiopharmaceutical uptake in bilateral occipital cortices with SPECT, indicating transhemispheric diaschisis across posterior cortical regions. Verley et al.^29^ demonstrated altered functional activation in the contralesional cortex with fMRI following muscimol-induced inactivation, supporting reversible functional diaschisis. Simard et al.^12^ showed seizure activity in remote regions using EEG 7–21 days after temporal lobe contusion, providing electrophysiologic evidence of interhemispheric diaschisis. Kaech et al.^19^ reported reduced frontal and parieto-occipital metabolism on PET up to 62 months post-TBI, reflecting persistent hypometabolism in regions distant from the primary lesion and therefore consistent with diaschitic processes.

Other diaschisis subtypes included white matter, subcortical, and visual diaschisis. Poudel et al.^25^ used connectome-based MRI modeling to predict patterns of Wallerian degeneration and gray matter atrophy up to 5 years post-injury. Schmitt et al.^26^ investigated visual diaschisis in an optic nerve injury model and found persistent suppression of local glucose metabolism in contralateral visual areas, unresponsive to cholinergic stimulation. These findings collectively illustrate the heterogeneity of diaschisis mechanisms in TBI and highlight the utility of multimodal imaging in tracking network-level disruptions across time.

On the contrary, diaschitic effects may not always be detectable on imaging and can present independently of radiographical abnormalities. Interestingly, Derakhshan^9^ described transcallosal diaschisis clinical findings (transient right-sided weakness and mild speech slurring) following post-traumatic epilepsy with subsequent subdural hematoma, with DWI showing diffusion restriction localized to the site of injury without contralateral extension.

### Functional outcomes

Functional outcomes following TBI varied across studies, showing a range of motor and cognitive impairments with differing recovery trajectories (Table 5). Ali et al.^8^ reported progressive left-sided hemiparesis accompanied by seizures and mental status decline in a patient with a severe TBI history five years prior, with clinical improvement after antiepileptic treatment. Boggs et al.^14^ observed hemiparesis, forelimb asymmetry, and motor coordination deficits early after injury, with partial recovery by 14 days post-TBI. Derakhshan^9^ described transient right-sided weakness and mild speech impairment following seizures, with full motor and partial speech recovery within a week. Drubach et al.^10^ reported spastic quadriparesis and persistent visual impairment six months post-injury despite partial improvement in the Glasgow Coma Scale (GCS).

**Table 5.** Functional Outcomes of Diaschisis

First author (year)	Motor deficits	Cognitive deficits	Recovery trajectory	Follow-up duration
Ali (2015)^8^	Left-sided hemiparesis, progressive seizures affecting left face, eye, limbs	Baseline vegetative state, recent decline in mental status	Initial worsening, improvement after antiepileptic treatment	Not specified (history of severe TBI 5 years prior)
Boggs (2024)^14^	Hemiparesis, forelimb asymmetry, increased paw faults	None reported	Partial improvement by day 2, near full recovery by day 14	14 days
Derakhshan (2009)^9^	Temporary right-sided weakness	Mild speech slurring post-seizure	Full motor recovery, partial speech recovery	1 week
Drubach (1994)^10^	Spastic quadriparesis (right side worse)	No significant cognitive deficits	Partial improvement in GCS, persistent visual impairment	6 months
Kaech (1996)^19^	Hemiparesis, ataxia, weakness	Executive dysfunction, memory impairment, poor concentration	Partial improvement, some residual deficits	6 months to 5 years
Lin et al. (2023)^22^	Not applicable (focus on cognitive scale)	No change	iPBM reduced incidence of CCD	3 months
Nishibe et al. (2010)^24^	Forelimb and hindlimb deficits (skilled reach, foot fault, contact placing)	Not assessed	Persistent motor deficits over 35 days, gradual improvement	35 days (key assessments at days 7, 14, 21, 28, 35)
Simard et al. (2025)^12^	Temporary impaired beam walk, rotarod	Sustained impaired novel object, Morris water maze, elevated plus maze	Full sensorimotor recovery; Persistent cognitive deficits	Up to 35 days
Verley et al. (2019)^29^	Impaired forelimb reaching	Not assessed	Improvement when contralesional cortex silenced at 1 week; no change or worsening at 4 weeks	Up to 4 weeks

GCS, Glasgow Coma Scale.

Longer-term motor and cognitive sequelae were also documented. Kaech et al.^19^ detailed hemiparesis, ataxia, and executive dysfunction persisting up to five years post-TBI, with some residual deficits despite partial recovery. Lin et al.^22^ found that while cognitive function remained stable following iPBM treatment, CCD incidence was reduced at three months. Nishibe et al.^24^ demonstrated persistent forelimb and hindlimb motor deficits up to 35 days post-injury in a CCI model, with gradual improvement noted in forelimb function by day 28. Simard et al.^12^ reported sustained memory and emotional impairments up to 35 days with minimal sensorimotor deficits.

Functional modulation of diaschisis effects was explored by Verley et al.,^29^ who showed impaired forelimb reaching on the injury side, with behavioral improvement when the contralesional cortex was temporarily silenced at one week, but not at four weeks post-injury. Overall, these findings highlight the heterogeneity of functional deficits post-TBI, as well as the potential for partial recovery and the influence of time and intervention on outcomes.

### Interventions

Several interventions targeting diaschisis mechanisms in TBI showed varying degrees of effectiveness (Table 6). One study demonstrated both reversal of diaschitic effects and functional benefit in animals: Verley et al.^29^ demonstrated that temporary silencing of the contralesional cortex using muscimol effectively restored ipsilesional motor control and improved forelimb reaching behavior at one week post-injury. However, this intervention was ineffective or detrimental when applied at four weeks, highlighting the importance of timing in modulating interhemispheric diaschisis.

**Table 6.** Interventions for Diaschisis

Author (year)	Intervention	Target mechanism	Outcome measures	Effectiveness
Deleglise (2018)^15^	MK-801 (NMDAR antagonist)	Blocks NMDA receptor-mediated excitotoxicity	Striatal neuron survival, dendritic pruning, calcium signaling	Fully prevented trans-synaptic degeneration and pruning
Deleglise (2018)^15^	Ifenprodil (GluN2B antagonist)	Blocks GluN2B-containing NMDA receptors	Striatal dendritic complexity, neuron death, survival rate	Completely reversed striatal pruning and neuron degeneration
Deleglise (2018)^15^	Tetrodotoxin (TTX)	Blocks cortical activity	Neuronal survival, dendritic complexity, striatal neuron degeneration	No significant effect when applied alone
Ihbe (2022)^16^	Isradipine (CaV1.3 blockade)	Modulates neuronal excitability in GABAergic interneurons	Electrophysiological recordings, calcium channel expression	Reduced post-traumatic hyperexcitability; no direct functional recovery reported
Lin et al. (2023)^22^	Intravascular photobiomodulation (iPBM)	Alleviates oxidative stress and mitochondrial dysfunction	Cognitive function, SPECT	Reduced incidence of CCD; no effect on cognition
Schmitt et al. (1998)^26^	Physostigmine (300 µg/kg)	Relieves injury-dependent suppression of local cerebral glucose use (LCGU)	Local cerebral glucose use in contralateral projection areas	No significant change in LCGU after treatment; suggested reversal of diaschisis between 2–9 days post optic nerve crush
Verley et al. (2019)^29^	Temporary silencing of contralesional cortex (muscimol)	Modulates interhemispheric diaschisis, restores ipsilesional motor control	Forelimb reaching behavior, fMRI signal changes	Effective at 1 week post-injury; ineffective/detrimental at 4 weeks

LCGU, local cerebral glucose utilization; TTX, tetrodotoxin.

Some interventions reversed structural diaschitic changes in *in vitro* models, though without reporting functional outcomes. Deleglise et al.^15^ demonstrated that MK-801, an N-methyl-D-aspartate (NMDA) receptor antagonist, fully prevented trans-synaptic degeneration and dendritic pruning by blocking NMDA receptor-mediated excitotoxicity. Similarly, Ifenprodil, a GluN2B-specific NMDA receptor antagonist, completely reversed striatal pruning and neuron degeneration.^15^ Although both interventions structurally reversed diaschitic effects, the studies did not assess whether these changes translated to functional improvement.

Other interventions reduced diaschitic findings but failed to demonstrate functional benefit. Lin et al.^22^ found in humans that iPBM reduced the incidence of CCD by alleviating oxidative stress and mitochondrial dysfunction, but this was not accompanied by cognitive improvement. Similarly, Ihbe et al.^16^ found in animals that Isradipine, a CaV1.3 calcium channel blocker, reduced post-traumatic hyperexcitability in contralateral GABAergic interneurons, indicating partial modulation of diaschitic excitability without corresponding behavioral recovery. In addition, Schmitt et al.^26^ showed in animals that physostigmine failed to enhance local cerebral glucose use (LCGU) in contralateral visual areas, indicating no recovery in visual pathway function despite partial reversal of diaschisis.

Finally, some interventions neither reversed nor reduced diaschisis. Deleglise et al.^15^ found in *in vitro* models that tetrodotoxin (TTX), a sodium channel blocker, had no significant effect on striatal neuron degeneration when used alone, suggesting no meaningful impact on diaschitic processes. However, functional outcomes were not assessed in this study, so no conclusions can be drawn regarding behavioral or clinical improvements.

## Discussion

This scoping review aimed to comprehensively map the existing literature on diaschisis in TBI, focusing on patterns of diaschisis types, underlying neurobiological mechanisms, clinical presentations, and the extent of intervention research. Our analysis revealed that crossed cerebellar and cortical-cortical diaschisis are among the most reported types in TBI, identified primarily through neuroimaging modalities such as PET, SPECT, MRI, and fMRI. Functional outcomes following diaschisis vary widely, with many studies reporting persistent motor and cognitive deficits but limited evidence of full recovery. Notably, interventional studies targeting diaschisis mechanisms remain scarce, with few demonstrating significant efficacy beyond early-stage experimental models. Important gaps include the underrepresentation of pediatric populations, a lack of longitudinal studies tracking diaschisis progression over time, and the paucity of clinical trials assessing targeted therapeutic approaches. Our findings underscore the need for focused research efforts on mild to moderate TBI in human populations, systematic intervention investigations, and large-scale clinical validation of imaging biomarkers correlated with functional recovery.

The predominance of CCD and cortical-cortical diaschisis in the literature likely reflects both the vulnerability of cortico-subcortical pathways to diffuse axonal injury and the relative ease of detection with current imaging techniques.^31^ The persistence of diaschisis-related hypometabolism or hypoperfusion observed in many studies suggests that diaschisis contributes to chronic network dysfunction beyond the primary lesion site, potentially impeding recovery. The variable timing of imaging and clinical assessments across studies may explain some inconsistencies in reported outcomes, with early interventions showing greater promise in modulating diaschisis effects. The lack of improvement with certain pharmacologic agents in later phases highlights the complex and dynamic nature of diaschisis, implicating both neuronal and glial mechanisms in its evolution. These findings collectively illustrate the heterogeneity of diaschisis mechanisms in TBI and highlight the utility of multimodal imaging in tracking network-level disruptions across time.

Compared with the extensive body of work on diaschisis in stroke, which often employs longitudinal PET or fMRI to correlate diaschisis with clinical recovery,^32^ the TBI literature is more fragmented and limited in scale. Stroke studies typically focus on acute to subacute phases with well-defined vascular lesions,^2^ whereas TBI studies must contend with heterogeneous injury patterns, mixed acute-chronic timepoints, and often multiple anatomically distributed sites of injury—making it more difficult to isolate and study diaschitic effects in TBI.^5^ Moreover, stroke research frequently includes larger sample sizes and standardized outcome measures,^33^ while TBI studies often vary widely in modalities used—ranging from FDG-PET to diffusion imaging—and in outcomes assessed, from motor deficits to cognitive impairments.

An important question in interpreting diaschisis in TBI is whether it primarily arises from white matter disruption, in contrast to stroke-related diaschisis. Evidence from this review suggests that in TBI, structural disconnection of long-range white matter tracts—such as corticopontine, transcallosal, and cortico-striatal pathways—is a primary upstream driver, leading to secondary dysfunction in anatomically intact gray matter regions. In stroke, although the primary lesion typically affects gray matter, diaschisis still reflects disrupted function in distant areas connected by white matter pathways. However, TBI is often characterized by diffuse, multifocal injury, making it more difficult to isolate diaschitic effects and more likely that they stem from widespread white matter disconnection, with downstream consequences such as neuronal hypoactivity, glial activation, and metabolic suppression.

Furthermore, compared with stroke, where the clinical consequences of diaschisis are relatively well-defined and closely linked to specific lesion patterns, the expression of diaschisis in TBI is markedly more heterogeneous. In patients with stroke, diaschisis has been associated with a range of clinical manifestations, including behavioral and cognitive changes, motor deficits such as hemiparesis, language impairments such as aphasia, and sensory disturbances affecting tactile and visual recognition.^32,34^ However, the clinical role of diaschisis remains a topic of ongoing research, particularly in TBI, where its correlation with behavioral changes—especially following subcortical injury—is not always well defined. As previously discussed, functional outcomes related to diaschisis in TBI appear more heterogeneous than in stroke, ranging from transient motor and speech impairments to persistent quadriparesis, visual deficits, executive dysfunction, and emotional disturbances, often with variable recovery trajectories.^9,10,12,19^

Overall, these findings highlight the heterogeneity of functional deficits post-TBI, as well as the potential for partial recovery and the influence of time and intervention on outcomes. However, it is important to note that most studies did not include comparator groups of TBI patients without evidence of diaschisis, making it difficult to isolate the specific contribution of diaschitic processes to functional deficits. Moreover, the functional consequences of diaschisis likely vary depending on lesion location, extent of network disruption, and individual patient factors, suggesting that there is no single clinical signature of diaschisis post-TBI.

These differences underscore the need for more systematic investigation into how diaschisis manifests and evolves in TBI, and how its clinical consequences may be modifiable through time- or intervention-sensitive mechanisms. In contrast to stroke, where targeted rehabilitation approaches for diaschisis have been extensively studied and increasingly integrated into clinical practice,^35,36^ the body of research investigating interventions for diaschisis in TBI remains sparse. Despite some promising preclinical findings, very few studies have explored therapeutic strategies aimed at modulating diaschisis in TBI, and even fewer have demonstrated meaningful functional improvements. This stark disparity highlights a significant gap in the literature and underscores the need for focused research to develop and validate interventions that address the complex, heterogeneous nature of diaschisis in TBI. Without such efforts, opportunities to improve recovery trajectories and patient outcomes may be missed.

This review uniquely broadens the scope to multiple diaschisis subtypes across diverse TBI populations, incorporating emerging imaging modalities and highlighting gaps in intervention research. Our inclusive approach emphasizes the need to integrate neuroimaging with clinical phenotyping and functional outcomes for a holistic understanding of diaschisis in TBI.

This review has limitations, including potential publication bias, exclusion of non-English and inaccessible studies, and heterogeneity in diaschisis definitions and measurement techniques. The variability in study designs, patient populations, and intervention protocols further complicates synthesis and generalizability. Future research should prioritize standardizing diaschisis terminology and imaging protocols to facilitate cross-study comparisons. Longitudinal neuroimaging studies are crucial to elucidate the temporal dynamics of diaschisis and its relationship to functional recovery, particularly differentiating cognitive from motor domains. Expanding clinical trials to test targeted therapies—pharmacologic, neuromodulatory, or rehabilitative—will be key to translating mechanistic insights into improved patient outcomes. A deeper understanding of diaschisis in TBI holds promise for advancing precision neurorehabilitation and tailoring interventions to individual pathophysiology, ultimately improving quality of life for survivors.

## Conclusion

This scoping review highlights the complex and multifaceted nature of diaschisis following TBI, demonstrating its widespread impact on brain networks and functional outcomes. Although diaschisis is increasingly recognized as an emerging contributor to post-TBI deficits, considerable gaps remain in our understanding of its mechanisms, progression, and response to targeted treatments—particularly in diverse patient populations and over extended recovery periods. Addressing these gaps through standardized imaging approaches, longitudinal studies, and rigorous clinical trials will be essential to harnessing the full potential of diaschisis research. Ultimately, advancing our knowledge of diaschisis in TBI promises to inform more precise, individualized therapeutic strategies that enhance recovery and long-term quality of life for patients.

## Abbreviations Used

18FDG-PET: Fluorine-18 Fluorodeoxyglucose Positron Emission Tomography
99mTc-HMPAO: Technetium-99m Hexamethylpropyleneamine Oxime
ACh: Acetylcholine
AEDs: Antiepileptic Drugs
BG: Basal Ganglia
CaV1.2: Calcium Voltage-Gated Channel Subunit Alpha1 C
CaV1.3: Calcium Voltage-Gated Channel Subunit Alpha1 D
CBF: Cerebral Blood Flow
CCI: Controlled Cortical Impact
CCD: Crossed Cerebellar Diaschisis
CFA: Caudal Forelimb Area
CO_2_: Carbon Dioxide
CT: Computed Tomography
DTI: Diffusion Tensor Imaging
DWI: Diffusion-Weighted Imaging
E/I: Excitatory/Inhibitory
EEG: Electroencephalography
EM: Electron Microscopy
FDG-PET: Fluorodeoxyglucose Positron Emission Tomography
FLAIR: Fluid-Attenuated Inversion Recovery
fMRI: Functional Magnetic Resonance Imaging
GABA: Gamma-Aminobutyric Acid
GABA-A: Gamma-Aminobutyric Acid Type A Receptor
GCS: Glasgow Coma Scale
GluN2B-NMDAR: Glutamate N-Methyl-D-Aspartate Receptor Subtype GluN2B
GSH: Glutathione
HI: Hypoxia-Ischemia
IHC: Immunohistochemistry
iPBM: Intracranial Photobiomodulation
LCGU: Local Cerebral Glucose Utilization
MAP-2: Microtubule-Associated Protein 2
MCA: Middle Cerebral Artery
MRI: Magnetic Resonance Imaging
MRA: Magnetic Resonance Angiography
NAA: N-Acetylaspartate
NDM: Network Diffusion Model
NMDA: N-Methyl-D-Aspartate
NMDAR: N-Methyl-D-Aspartate Receptor
O_2_: Oxygen
P14: Postnatal Day 14
PARP: Poly (ADP-ribose) Polymerase
PCA: Principal Component Analysis
PET: Positron Emission Tomography
PRISMA-ScR: Preferred Reporting Items for Systematic Reviews and Meta-Analyses Extension for Scoping Reviews
PV+: Parvalbumin-Positive
RFA: Rostral Forelimb Area
SPECT: Single Photon Emission Computed Tomography
SST: Somatostatin
T2-RARE: T2-Weighted Rapid Acquisition with Relaxation Enhancement
TBI: Traumatic Brain Injury
tlCont: Temporal Lobe Contusion
TTX: Tetrodotoxin
UAE: United Arab Emirates
UK: United Kingdom
USA: United States of America
VGCC: Voltage-Gated Calcium Channel
WFA: Wisteria floribunda agglutinin
